# Development and validation of a rapid HPLC- fluorescence method for simultaneous determination of venlafaxine and its major metabolites in human plasma

**Published:** 2010

**Authors:** Y. H. Ardakani, A. Foroumadi, M.R. Rouini

**Affiliations:** 1 Biopharmaceutics and Pharmacokinetics Division, Department of Pharmaceutics, Faculty of Pharmacy; 2 Drug Design & Development Research Center, Tehran University of Medical Sciences, Tehran, Iran

**Keywords:** Venlafaxine, Metabolite, Pharmacokinetics, HPLC

## Abstract

**Background and the purpose of the study:**

To develop a simple, rapid and accurate HPLC method for the measurement of the venlafaxine and its main metabolites, *O*-desmethylvenlafaxine and *O*,*N*-didesmethylvenlafaxine in pharmacokinetic studies and therapeutic drug monitoring.

**Method:**

Chromatographic separation was achieved with a ChromolithTM Performance RP-18e 100 mm×4.6 mm column equipped with a Fluorescence detectore (λ_ex_ 200 nm/λ_em_ 300 nm) The mobile phase of methanol:water (35:65, v/v) adjusted to pH 2.5 by phosphoric acid was passed through the column in an isocratic mode at flow rate of 2 ml/min. The sample preparation involved a simple, one-step, extraction with ethyl acetate.

**Results:**

The calibration curves were linear in the concentration range of 1-300 ng/ml for all analytes (r2>0.998). The lower limit of quantification was 1 ng/ml for all analytes. Within and between day precisions in the measurement of quality control (QC) of samples were in the range of 1.8-14.1% for all analytes.

**Conclusion:**

The developed procedure was used to assess the pharmacokinetics of venlafaxine and its main metabolites following oral administration of 75 mg venlafaxine to a healthy subject.

## INTRODUCTION

Venlafaxine (VEN) (1-[2-(dimethylamino)-1-(4- methoxy-phenyl) ethyl] cyclohexanol hydrochloride) is a novel antidepressant, which enhances noradrenergic and serotonergic neurotransmission through inhibition of noradrenaline and serotonin reuptake. It also to a lesser extent inhibits, dopamine reuptake. VEN shows no MAO (Monoamine oxidase) inhibitory activity and has no affinity for histamine, α_2_ or β-adrenergic and muscarinic spectrometry has high sensitivity but is expensive and receptors (1). On the basis of clinical trials, this drug appears to lack many side effects associated with tricyclic antidepressants.

**Figure 1:**
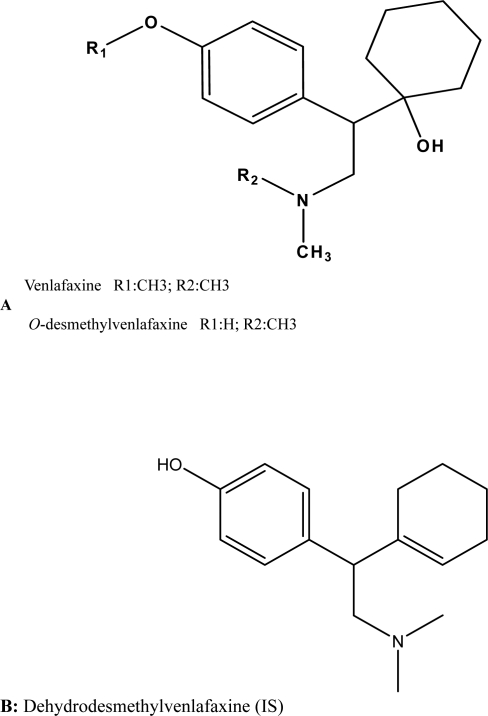
Chemical structure of (A) Venlafaxine and its metabolites and (B) Dehydro Desmethylvenlafaxine as internal standard.

In humans, VEN is absorbed almost completely (92%) after oral intake and undergoes extensive metabolism in the liver (Figure 1). About 1% of VEN is desmethylated to *N*-desmethylvenlafaxine (NDV);

16% becomes *O*, *N*-didesmethylvenlafaxine (DDV) and 56% is metabolized to *O*-desmethylvenlafaxine (ODV) (2). Among all these metabolites, ODV is pharmacologically active with higher concentrations and longer half-life than the parent compound (4-9 hrs versus 11–13 hrs) and significantly contributes to the therapeutic effects of VEN. Therapeutic plasma levels of VEN usually range from 30 to 200 ng/ml, while the corresponding levels of ODV are in the range of 50– 500 ng/ml (3).

Several methods can be found in the literature for monitoring the plasma levels of VEN and ODV in biological samples (4–11). The reported high performance liquid chromatography (HPLC) methods have used mass spectrometric (4–6), fluorimetric (7–9), and UV detection techniques (10, 11). Electrospray not commonly available in most analytical laboratories (4–6). Besides, the reported limit of quantification for VEN (5, 6) is almost the same as fluorimetric (9) or higher than coulometric reported methods (12).

Other published methods are based on the preliminary extraction of analytes from the samples using solid phase (5, 9) (13) or organic solvents (7, 8) (10, 11). However, large (1 ml) sample volume (10, 11), high chromatographic temperature (60°C) (8), relatively high limit of quantification (values in the range of 5–50 ng/ml) (8–12) and/or long analysis time (≥10 min) (7–12) are of those disadvantages.

To our knowledge, there is only one validated HPLC-MS report for simultaneous determination of VEN and its three metabolites (4), and all other published methods have only determined VEN and ODV as major metabolite. Even though DDV is not an active metabolite, it supplies a major metabolite of VEN which makes it important for pharmacokinetic studies.

The objective of this study was to reduce the duration of analysis considerably while maintaining the sensitivity required for the detection of these compounds in their therapeutic range by a newly developed monolithic HPLC column. Therefore a simple, rapid and accurate HPLC method was developed for the measurement of the VEN, ODV and DDV in pharmacokinetic study of VEN as well as therapeutic drug monitoring (TDM).

## EXPERIMENTAL

### 

#### Chemicals and reagents

Pure powder of Venlafaxine, *O*-desmethylvenlafaxine, *O,N*-didesmethylvenlafaxine (>98%) were provided by Osvah Pharmaceutical Co. (Tehran, Iran). The internal standard of *O-*desmethyldehydrovenlafaxine (Figure 1) was synthesized by the literature method (13). HPLC-grade methanol and analytical grade ethyl acetate and phosphoric acid (85%), were supplied by Merck (Darmstadt, Germany). Water used in all experiments was of Direct-Q® quality (Millipore, France).

#### Preparation of O-desmethyldehydrovenlafaxine as internal standard

To a stirred solution of 1-(2-(dimethylamino-1- (4-methoxyphenyl) ethyl)-cyclohexanol (venlafaxine free base) (1387 mg, 5 mmols) in CH2 Cl_2_ (30 ml) cooled to -10 °C, under N_2_ was added slowly 10 ml (10 mmol) of 1 M solution of BBr in CH_2_ Cl_2_ over a period of 5 min. The reaction mixture was warmed to 0 °C and stirred for 3 hrs. During this period of time a gummy precipitate was formed. To the stirred mixture, was slowly added 20 ml of 2.5 N sodium hydroxide solution, allowed to warm to room temperature and stirred for 3 more hrs. The organic solvent was then removed by evaporation under reduced pressure. The aqueous layer having a pH of 13-14 was extracted with ethyl acetate (3×10 ml) and combined EtOAc extracts were dried over anhydrous MgSO4, filtered and evaporated in vacuum to afford a white solid, which was mainly contained *O*-desmethylvenlafaxine on the basis of physical and spectroscopic data consistent with literature values (12) and a minor product. The minor product which its structure according to the spectroscopic data was assigned to be the dehydrated product (*O*-desmethyldehydrovenlafaxine) separated on silica gel (Merck 230-400 mesh) column using CH_2_ Cl^2^ –CH_3_ OH (20:1, v/v) as mobile phaseThe purity of the internal standard was studied by the abovementioned HPLC method, except for the composition of mobile phase which was changed to methanol: water (25:75, v/v) for further improvement in peak resolution.

#### Apparatus and chromatographic condition

Low-pressure HPLC pump (K-1001) was connected to a fluorescence detector [excitation wavelength (λ_ex_) 200 nm/emission wavelength (λ_em_) 300 nm] an online degasser, all from Knauer (Berlin, Germany). A Reodyne model 7725i injector with a 100 µl loop was used. The data was acquired and processed by means of ChromGate chromatography software (Knauer, Germany).

Chromatographic separation was achieved by a ChromolithTM Performance RP-18e 100 mm×4.6 mm column (Merck, Darmstadt, Germany) protected by a ChromolithTM Guard Cartridge RP-18e 5 mm×4.6 mm at room temperature. For the mobile phase, a mixture of methanol: water (35:65, v/v) adjusted to pH 2.5 by phosphoric acid (final acid concentration about 1.5 mM), was delivered in isocratic mode at 2 ml/min flow rate.

### Sample preparation

#### Standard solutions

Stock solutions of the analytes (1 mg/ml) were prepared by dissolving suitable amount of each pure substance in methanol. Intermediate pooled stock standard of all compounds in concentrations of 10 µg/ml were prepared using mobile phase as solvent. Standard solutions were prepared freshly every day.

#### Preparation of calibration standards

From stock solutions of VEN, ODV and DDV (10 µg/ml) in mobile phase, standard solutions were prepared in human drug free plasma obtained from healthy volunteers as diluent. The calibration curve was performed with standard solutions of the final concentration of 1-300 ng/ml for VEN, ODV and The DDV in human plasma respectively.

#### Extraction procedure

All analytes of interest were extracted from plasma by liquid–liquid extraction (LLE). The conditions consisted of mixing 200 µl of plasma with 50 µl of Internal Standard (IS, 1 µg/ml) and 50 µl of NaOH (2N) in a 2 ml Eppendorf polypropylene tube and then extracting with 1.2 ml of ethyl acetate. After vertical agitation (15 min) and centrifugation (14500×g, 2 min), the upper organic layer was transferred into a conical tube and evaporated under a gentle stream of air. The dried extract was re-constituted in 120 µ1of the mobile phase and a 100 µl aliquot was injected onto the HPLC system.

#### Application of the method

The study protocol was approved by the Ethics Committee of Tehran University of Medical Sciences. After obtaining written informed consent, to a healthy subject who was fasting overnight, was administered a single dose 75 mg of VEN tablet (Efexor, Wyeth-Ayerst, Canada). Peripheral venous blood samples were taken from the subject at predetermined intervals and plasma samples were stored at -20 °C until analysis.

## RESULTS

### Method validation

#### Selectivity

**Figure 2:**
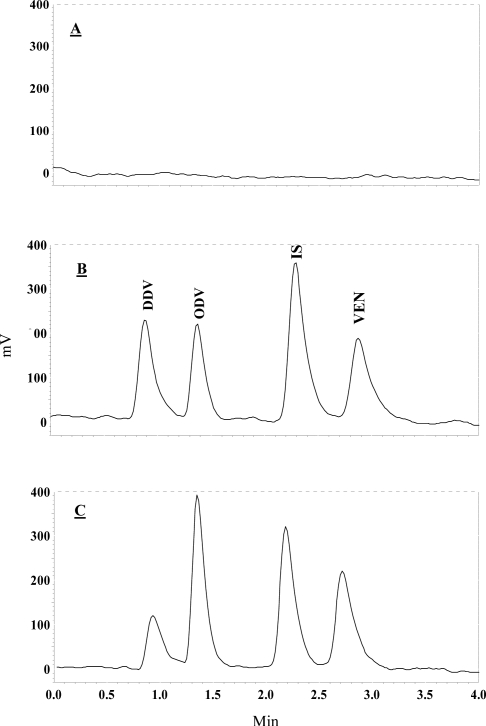
Chromatograms of (A) blank human plasma, (B) plasma spiked with 125 ng/ml of VEN, ODV and DDV and (C) plasma of the volunteer 2.5 hrs after single oral administration of 75 mg venlafaxine (VEN: 121.3 ng/ml, ODV: 268.7 ng/ml and DDV: 47.0 ng/ml).

The chromatograms of analytes under study by the present assay method are presented in Figure 2.

Selectivity was indicated by the absence of any endogenous interference at retention times of peaks of interest as evaluated by chromatograms of control human plasma (using more than nine different blank plasma samples) and plasma spiked with compounds. Under the stated conditions, DDV, ODV, IS and VEN were eluted at approximately 0.9, 1.3, 2.2 and 2.7 min respectively with less than 3% of RSD for retention times of these analytes (Table 1). The system suitability factors were calculated according to the U.S. pharmacopeia (14) and the results are summarized in Table 1. No interference was observed with commonly co-administered medication with VEN either because of lack of the fluorescence emission or very short/long retention time in the developed system.

**Table 1:** System suitability parameters calculated for DDV, ODV, IS and VEN.

Capacity Factor (K')	Plate count (N)	Asymmetry factor (Af)	Resolution (R_s_)	Ret. Time(RSD) (min)	Analyte
3.5	2916	1.1	1.9	2.7 (1.2)	**VEN**
2.7	1936	1.2	1.6	2.2 (2.9	**ODV**
0.5	576	1.2	1.2	0.9 (1.5)	**DDV**
1.2	676	1.2	3.4	1.3 (2.6)	**IS**

#### Linearity

The linearity of the relationship between peak area ratios and corresponding concentrations (which were required in this study) were demonstrated by the correlation coefficients obtained for the regression lines. Table 2 presents the linearity data from the analysis of all analytes. Each concentration level of the curve is based on data of three separate runs. The linearity analysis showed coefficients of correlation greater than 0.998 for all compounds (Table 2).

**Table 2:** Linearity data from the analysis of venlafaxine and it's two main metabolites.

Intercept (R.S.D)	Slope (R.S.D)	r^2^	Linearity
0.0002(5.6)	0.0076(2.6)	0.9995	**VEN**
0.0124(6.8)	0.0041(1.7)	0.9984	**ODV**
0.0095(7.1)	0.0039(3.1)	0.9986	**DDV**

*r^2^=Coefficient of correlation

#### Accuracy, precision and recovery

Between- and within-day accuracy and precisions of the method were determined for each compound according to the FDA guidance for bioanalytical method validation (15). Five replicate spiked samples were assayed between- and within-day at four different concentrations of each analyte in plasma. Accuracy was calculated as deviation of the mean from the nominal concentration. Precision was expressed as the relative standard deviation of each calculated concentration. Average recovery of each compound was determined by comparing AUC obtained after injection of the processed QC samples with those achieved by direct injection of the same amount of drug in mobile phase at different concentrations (five samples for each concentration level). The results are reported in Table 3. Accuracy studies of the plasma showed acceptable values (92.7-108.1%) for both between- and within-day studies (n=5). The values of within and between-day precision were below 14.1% for all samples. The absolute recoveries of all analytes from plasma samples were between 85.0 and 95.7% as shown in Table 3.

**Table 3:** Recovery, between and within-day accuracy and precision of the HPLC method for the determination of venlafaxine and its two main metabolites (n=5).

	Concentration(ng/ml)	Recovery		Between-day		Within-day	
		
		R.S.D	%	R.S.D	Accuracy	R.S.D	Accuracy
**VEN**	2.5	10.1	91.2	7.9	101.8	14.1	102.7
	10	11.9	94.6	2.2	99.4	10.1	100.7
	25	7.8	93.7	2.8	92.7	7.8	90.1
	50	9.1	95.7	1.8	97.7	3.7	96.2
**ODV**	2.5	12.5	85.7	9.5	103.0	11.3	108.1
	10	12.1	90.7	1.8	107.8	9.1	105.4
	25	10.8	89.4	5.0	104.3	7.6	106.0
	50	11.1	91.6	2.2	97.9	10.1	94.9
**DDV**	2.5	13.2	85.0	8.7	100.4	12.1	104.6
	10	14.1	86.4	7.2	99.4	9.5	106.1
	25	10.9	88.9	6.1	101.1	10.4	100.4
	50	13.7	88.8	4.8	98.7	8.4	101.1

#### Lower Limit of quantification

The lower limit of quantification (LLOQ) was defined as the lowest analyte concentration, which can be determined with an accuracy and precision <20% (15). The calculated LLOQ values were 1 ng/ml with RSD and accuracy values between 14.2-16.7% and 98.7-104.0% for of all analytes respectively.

#### Stability

Stock solutions of VEN, ODV and DDV in methanol were stored at 4°C for 2 months and all analytes appeared to be stable. The samples spiked with the analytes were stable after storage at -20°C over 3 months. The stability of wet and dry extracts were examined with three replicate spiked quality control samples and no significant degradation were observed for IS and all analytes. No degradation in plasma samples subjected to three cycles of freeze–thaw was observed. A complete stability study for plasma samples has been reported previously (4, 16).

#### Application of the method

**Figure 3:**
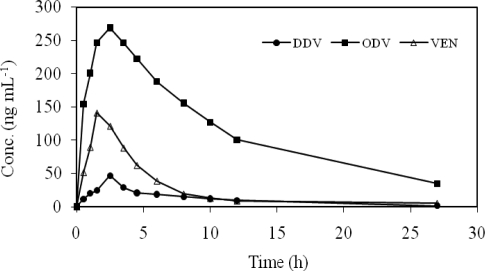
Concentration–time profile of VEN, ODV and DDV after administration of 75 mg of single oral dose of venlafaxine to one volunteer.

The described assay procedure was applied successfully to the pharmacokinetic study of the VEN and its metabolites in one healthy volunteer. Plasma concentration-time courses of VEN, ODV, and DDV following a single oral dose of VEN are shown in Figure 3. After 0.5 hrs of administration, the ODV concentration in plasma considerably exceeded the plasma concentration of parent compound. However the maximum concentration occurred nearly 1 h later in the plasma. In summery, the maximum plasma concentrations were 141.2, 268.7 and 47.0 ng/ml which were observed nearly 1.5, 2.5 and 2.5 hrs after administration of VEN, ODV and DDV respectively. The elimination half- lives of VEN, ODV and DDV were calculated about 5.1, 8.2 and 5.4 hrs respectively.

## DISCUSSION

Long run time in most of HPLC methods may be the result of limited flow rate of routine HPLC columns which might be resolved by using recently developed monolithic HPLC columns such as ChromolithTM. These types of columns have a biporous structure which results in higher porosity compared to usual columns. Therefore, they could be used with high flow rates without significant loss of performance or other limitations due to increased pressure. Consequently these types of columns achieve faster separation than those of conventional HPLC columns and shorter runs will usually cause better sensitivities.

The described method has been established as a rapid analytical tool in pharmacokinetic, therapeutic drug monitoring (TDM) or toxicological studies requiring short retention time, high precision, sensitivity and small volumes of plasma for analysis. This method also provided simultaneous determination of VEN and its two main metabolites. Although no clinical importance has been described for the inactive metabolites, measurement of these types of analytes in human plasma may be helpful in better understanding of the metabolism of the parent compounds. Besides, DDV has very similar structure and hence lipophilicity to ODV and reaches to a considerable fraction of the active metabolite (∼30%). Therefore, any interference between ODV and DDV (because of almost similar structure) may cause significant error in measurement of the active metabolite (ODV) concentration. As it was mentioned in introduction, Liu et al*.* have reported an HPLC-MS method which separates VEN, ODV, DDV as well as NDV (4). Since only a minor amount of VEN is metabolized to NDV (about 1%), in the current investigation this metabolite was not detected and analyzed in human plasma.

Although not resolving DDV, Vu et al*.* have reported an HPLC method with a tedious and time consuming extraction procedure (overnight freezing of the samples at -20 °C) reaching a maximum recovery of 68% for ODV using hexane/isoamyl alcohol as extraction solvent (7). In addition to low recovery for ODV (52%), the method published by Waschgler et al. suffers from inappropriate sensitivity (around 20 ng/ml) and high chromatographic temperature (60 °C) (8). Recently Mandrioli et al*.* have published a sensitive HPLC method using SPE as extraction procedure (9), however this method has relatively long run time and unable to resolve DDV.

Application of the monolithic HPLC column (ChromolithTM) allowed us to resolve four analytes (including IS) more rapidly with a total run time of less than 3.5 min producing very sharp peaks with lower limit of quantification (LLOQ) of 1 ng/ml.

In this study a specific internal standard was synthesized with maximum structural similarity which resulted to a shorter overall chromatographic run time. As it is clear from Figure 2, the IS is eluted between ODV and VEN with a retention time of 2.2 min. In this study citalopram which has been used as IS in many previously published VEN analytical methods was also examined. Although results show that this compound could also be used as IS but the run time might lengthen up to 4.5 min. The use of ethyl acetate (safe and popular solvent) as extracting solvent also produced a complete recovery (almost 90%) for all analytes with a very clean and interference free baseline.

In the last few years, TDM has acquired more importance in antidepressant therapy, especially when abnormal metabolism or low compliance is suspected, or in the case of polypharmacy. Therefore, a simple analytical procedure based on a rapid one step extraction and a total run time of less than 3.5 min allows the possibility of determination of at least 50 samples a day in TDM or in pharmacokinetic studies.

## CONCLUSION

A sensitive, accurate and precise bioanalytical method involving a simple liquid–liquid extraction of samples was developed and validated for determination of VEN and its principal metabolites. This procedure may be recommended for pharmacokinetic and forensic studies as well as for therapeutic drug monitoring.
